# The cycle of infectious fever – how it affects bacterial infections

**DOI:** 10.3389/fcimb.2026.1866500

**Published:** 2026-07-22

**Authors:** Kinga Kramarczyk, Piotr Schmidt, Karina Krasna, Weronika Jasińska, Anna Jarzab

**Affiliations:** Department of Immunology of Infectious Diseases, Hirszfeld Institute of Immunology and Experimental Therapy Polish Academy of Sciences, Wroclaw, Poland

**Keywords:** bacteria, fever, immune response, infection, pyrogens

## Abstract

Fever is one of the human body’s most effective strategies in fighting infections. However, its precise role in eliminating pathogens, specifically the influence of heat on the bacterial cells on the mechanistic level under febrile temperatures has never been fully delineated. The concept of fever, as the immune system’s first line of defense against pathogens involves two main components: the inflammatory response and the heat, which stimulates the host’s immune cells and affects pathogens. This article summarizes independent findings, grouping the development of fever into a circular model, starting with pathogen invasion and the release of bacteria’s exogenous pyrogens, continuing through the host immune response, and debating on the impact of fever on numerous human pathogens. This model focuses broadly on processes occurring within bacteria, particularly pathogen adaptation and the detrimental effect of heat released during fever on pathogen death. We also discussed the impact of commonly used antipyretics, such as acetaminophen, ibuprofen, and aspirin, on the overall outcome of infection and provided a framework within which new concepts and findings in this field can be developed.

## 1 Introduction

Fever is a key symptom of the body’s response to various external or internal stressors, infections, or injuries and is an important component of the innate immune response. In humans, elevated body temperature accompanying fever is often considered as an unwelcome symptom, or even a direct contributor to the disease. The cultural features of fever encompass how different societies perceive, fear, and treat elevated body temperatures. While biological symptoms including shivering, sweating, and malaise are universal, cultural attitudes range from viewing fever as a beneficial defense mechanism to fearing it as a harbinger of permanent brain damage. Therefore, in modern society, even mild fever is considered as a very negative sign and is often immediately treated with antipyretics or antibiotics. Febrile temperatures can be divided into several groups and can be classified as low-grade (37.3 to 38.0 °C), medium-grade (38.1 to 39.0 °C), high-grade (39.1 to 41 °C) and hyperthermia/hyperpyrexia when the temperature rises above 41 °C (Balli et al., 2025). Physicians routinely prescribe fever reducing drugs and/or antibiotics as soon as a patient’s temperature reaches 39 °C (de’Angelis et al., 2025). Similarly, at home, people often use commonly available antipyretics such as paracetamol or ibuprofen to reduce high body temperatures (Sarrell et al., 2006; Evans et al., 2015).

On the other hand, when microorganisms are exposed to temperatures exceeding their maximum growth limits, their cell viability rapidly declines. An increase in the body temperature of 1-4 °C helps to fight infections caused by many different pathogens. For example, febrile temperatures of 40–41 °C facilitate the reduction in the replication rate of poliovirus in mammalian cells for over 200-times and increase the susceptibility of Gram-negative bacteria to serum-induced lysis (Evans et al., 2015). However, the specific critical step, the primary event that triggers pathogen’s death under fever, has been a subject of debate. Two prevailing hypotheses have historically dominated the field. The first one is based on the membrane damage and refers to thermal stress which disrupts the cytoplasmic membrane, causing leakage of intracellular components (Brock, 1967; Russell and Harries, 1968). The second one focuses on the denaturation of ribosomes, enzymes or other cytoplasmic molecules and degradation of crucial cellular components, such as nucleic acids as the primary lethal event (Stephens and Jones, 1993; Tolker-Nielsen and Molin, 1996). However, it has never been precisely determined what temperature is crucial for pathogens to be thermally inactivated, and especially under what circumstances the heat associated with fever affects the death of the pathogen. It is also clear that both the host’s body temperature and the specificity of the immune response depend greatly on the pathogen causing the infection. Literature data demonstrate numerous examples of the positive impact of infectious fever on combating viral infections. Similar data regarding the effect of fever on bacteria are much more scattered and incomplete. Therefore, focusing research attention in this direction would be extremely useful in the context of examining the impact of fever-related temperature on the course of bacterial infection.

The influence of the immune response on combating bacterial infections is unquestionable and the role and the mechanisms of actions of both the innate and adaptive immune responses have been extensively studied and described. The literature provides information on a wide range of acquired bacterial processes occurring inside the bacterium during the development of fever. Unfortunately, the comprehensive mechanisms of cell killing by temperature are not fully understood, and the impact of temperature alone versus its synergistic effect with the immune system remains unclear. This is a vast area of ​​research constituting an important gap, and its exploration would elucidate the potentially beneficial role of fever in combating infections. Given that in many cases, inflammation and accompanying fever, unaided by therapeutic agents, allow for complete recovery from bacterial infections. Therefore, it would be extremely valuable to identify the key factors responsible for bacterial cell death, and in particular, to uncover the role of inflammation-induced temperature in triggering the mechanisms responsible for bacterial cell death during fever. In this review, we have gathered available findings on the entire process of interaction between the human immune response and bacterial cell defense mechanisms during fever. We have addressed aspects that are currently well understood and described, and identified gaps in our knowledge of the entire process.

## 2 The general influence of fever on the host and the pathogens

When a high fever progresses to uncontrolled hyperthermia, a condition when the core body temperature exceeds 40 °C and the body’s internal thermostat fails completely, it can lead to multiple organ failure (Iba et al., 2025). One of the reasons for high fever might be associated with septic shock during severe bacterial infections which is a life-threatening medical emergency. Septic shock occurs when the monocytes, macrophages, and neutrophils are hyperactivated and interact with the endothelium leading to further production of cytokines, proteases, kinins, reactive oxygen species, and nitric oxide. This leads to the microvascular injury of the endothelium and often activates the coagulation and complement cascades, which further exacerbate the vascular injury, leading to capillary leakage. In consequence this cascade is responsible for the clinical signs of sepsis (Doman et al., 2023). Moreover infectious fever can be a symptom of hyperinflammation, which indicates an overactive immune system, frequently called a cytokine storm. Under severe hyperinflammation, the body releases excessive inflammatory proteins, which can cause multi-organ damage, low blood pressure, extreme fatigue, and severe breathing difficulties (Nie et al., 2025). In fact, high fevers leading to those dangerous consequences are very rare. High temperatures are most dangerous for infants under 3 months old, young children between 6 months and 5 years of age prone to seizures, people with chronic heart or lung conditions, and immunocompromised patients (Alsabri et al., 2025). Immunocompromised individuals on chemotherapy, with HIV/AIDS, or taking immunosuppressants face severe complications from even low-grade fevers, extreme temperatures above 41 °C risk damaging internal organs, and rapid temperature spikes can trigger seizures in young children. Even, the awareness of such complications is crucial, we should be aware that fever is the body’s natural defense against infection and lowering body’s temperature should be well balanced, since the general fear of high fevers leads to excessive use of antipyretics and antibiotics. On the other hand, numerous studies suggest that warming the body is associated with a shorter duration of infection and improved host survival. For example, rhinoviruses, the main cause of the common cold, prefer lower temperatures, and temperatures of 37-38 °C and higher significantly limit their ability to replicate and survive. Human body temperature (37 °C) is higher than optimal for the virus, making reproduction less efficient. Higher temperatures (e.g., a fever of 38 °C+) weaken the virus, helping the immune system (Foxman et al., 2015). Positive influence of elevated temperatures were also shown for bacteria *in vitro*. It was shown that cultures of methicillin-resistant *Staphylococcus aureus* were more susceptible to the inhibitory effects of methicillin on growth and peptidoglycan synthesis at fever-like temperatures at 40 °C. Conversely lower temperatures (30 °C) enhance resistance making peptidoglycan synthesis and growth more resistant to methicillin. This effect was associated with lower production of penicillin-binding protein 2 (PBP2) at 40 °C (Madiraju et al., 1987).

An excellent example indicating the importance of temperature influence on pathogens, are the bird derived viruses. The body temperature of birds (40-42 °C) corresponds to the temperatures reached during the later stages of fever in humans. One of the best-known viruses infecting birds is the influenza A virus, which can be spread between various animal hosts. The virus replicates in the gastrointestinal system of birds at a temperature of 40-42 °C, whereas seasonal human influenza A virus replicates mainly in the colder upper respiratory tract (around 33 °C) and is less tolerant to temperatures around 40 °C. The study compared the human influenza virus with a modified variant containing avian-like features. The focus was on the PB1 gene, which encodes one of the viral polymerase proteins, because avian variants of this gene promote viral replication at higher temperatures. It was shown that in mice whose body temperature was artificially raised to a fever-like level, the human influenza virus caused a milder course of the disease. Conversely, the modified virus, which carried avian-like features, showed resistance to the elevated temperature and still caused severe infection. These results suggest that some viruses transmitted from birds to mammals may remain dangerous because they are partially adapted to higher temperatures, which in humans represent an important natural defense mechanism against infection (Turnbull et al., 2025).

Similarly, a relatively new variant of the avian influenza virus is the A (H5N1) virus from clade 2.3.4.4b, which has spread rapidly worldwide since 2020, causing mass deaths of wild birds and poultry. A key feature of this virus is its ability to reassort its genome - this is the exchange of RNA segments between different influenza viruses infecting the same cell, which can lead to the emergence of new, potentially more dangerous variants. According to WHO data, transmission of the virus among animals continues, while the number of human infections remains low. However, recent studies indicate, that infections in mammals and transmission between mammals are becoming increasingly common, which may create new opportunities for the virus to adapt to humans (Capelastegui and Goldhill, 2025; Peacock et al., 2025).

Furthermore, *Campylobacter* is a bacterium that causes infections in humans and is associated with birds. *Campylobacter* grows optimally at a temperature around 42 °C. The main reservoir of this bacterium is poultry, which is why *Campylobacter* effectively colonizes the digestive tract of birds, where temperatures reach 41-42 °C. However, once transmitted to humans, it can cause gastrointestinal infections (El-Saadony et al., 2023).

These examples clearly describe the meaning of elevated temperature in combating viral and bacterial infections. Their reservoirs in different hosts, like those originally coming from birds show that the temperature plays a role in keeping these pathogens under control and preventing their further expansion. Furthermore, dating back to ancient times (Maloney and Duffy, 2024), there are evidences suggesting that fever may be our body’s strategy for accelerating defense against a wide variety of diseases. The general opinion points to the enhanced positive effects of heat on the development of the host immune response and its harmful effects on the pathogens themselves.

## 3 Internal and external pyrogens affect human immune response and trigger fever development

Elevated temperature affects both the host organism and the pathogen. The standard human body temperature is considered to be 37 °C, but it can vary by up to 1 °C. Fever arises as a result of the action of pyrogens, substances that elevate body temperature as part of the host’s defense to microbial components. For instance, temperatures including fever (40-41 °C) accelerate serum-induced lysis of Gram-negative bacteria and enhance action of the immune system including secretion of IL-6, which drives the growth of body temperature and coordinates migration of lymphocytes to lymphatic organs, e.g. spleen or lymph nodes (Evans et al., 2015; Walter et al., 2016).

### 3.1 Internal pyrogens

Pyrogenic cytokines (IL-1, IL-6 and TNF-α) are internal pyrogens, secreted by host’s organism and they play key roles in the induction of fever. In addition, IL-6 has been identified as essential for maintaining it. Mice lacking IL-6 or blocked with anti-IL-6 antibodies do not develop fever, despite the presence of the other two cytokines whereas injection of IL-6 into the mouse brain restores the febrile response, while injection of IL-1 does not have the same effect (Chai et al., 1996; Cartmell et al., 2000). Internal pyrogens are often secreted as a response of host’s organism to exogenous pyrogens, derived from pathogens.

### 3.2 Exogenous pyrogens

Exogenous pyrogens originate outside the body, most commonly from components of microorganisms such as bacterial lipopolysaccharide (LPS), lipoteichoic acid (LTA), or flagellin. These molecules do not act directly on the thermoregulatory center in the hypothalamus; instead, they bind to Pattern Recognition Receptors (PRRs), such as Toll-like receptors (TLR) on host immune cells, initiating an inflammatory response (Dinarello, 2004). As a result of Pathogen-Associated Molecular Patterns (PAMPs) recognition, macrophages and dendritic cells release PGE2 – main pyrogenic mediator, and other pro-inflammatory factors, such as IL-1, IL-6 and TNF-α, which act on the hypothalamus to increase body temperature and induce fever. In response to infection, PGE2, IL-1, IL-6 and TNF-α induce fever and activate additional immune cells – neutrophils and T lymphocytes (Evans et al., 2015). Pyrogenic toxins produced by streptococci and staphylococci are important PAMPs, which act as superantigens. These are strong T-cell mitogens that bind simultaneously to Major Histocompatibility Complex (MHC) molecules on antigen-presenting cells (APC) and to T-cell receptors. This leads to a massive release of pro-inflammatory cytokines, including IL-1β and IFN-γ (Cavaillon, 2018).

Flagellin, a protein derived from the flagella of both Gram-negative and Gram-positive bacteria, also exhibits pyrogenic activity and is recognized by the Toll-like receptor 5 (TLR5). Upon binding of flagellin, the receptor becomes activated, initiating a signaling cascade that leads to the activation of the nuclear factor NF-κB, a key transcription factor that regulates the expression of numerous genes involved in inflammation, immune responses, and cell survival (Hayashi et al., 2001).

Another strongly pyrogenic bacterial component is CpG-DNA, which contains unmethylated CpG motifs characteristic for bacterial genomes. Studies have shown that CpG-DNA rapidly activates immune responses leading to fever in a dose-dependent manner.

CpG-DNA acts primarily through TLR9. This activation leads, among other effects, to the production of IL-6, which is responsible for stimulating prostaglandin E2 synthesis in the hypothalamus and regulating inflammatory processes (Kozak et al., 2006).

Among different microbial pyrogens, one of the most widely studied and classical models for investigating fever induction is lipopolysaccharide (LPS), a component of the outer membrane of Gram-negative bacteria. Administration of exogenous LPS to rodents reliably triggers an inflammatory response and an increase in body temperature, closely mimicking the febrile reaction observed during bacterial infection in humans (Roth and Blatteis, 2014).

LPS acts as a potent trigger for the innate immune system by binding to Toll-like receptor 4 (TLR4) on immune cells. This process starts from LPS-binding protein (LBP), which transports LPS to the surface of immune cells, e.g. monocytes/macrophages and neutrophils, where it binds to CD14 membrane protein. Then through TLR4 and medullary differentiation protein 2 (MD2), which form a protein complex. This event initiates a cascade of intracellular signaling, including molecules such as myeloid differentiation factor 88 (MyD88), IL-1R-associated kinase (IRAK) and IRAK2, and TNF receptor-associated factor 6 (TRAF6) leading to the activation of transcription factor nuclear factor kappa B (NF-κB), which in turn triggers the production of inflammatory cytokines and chemokines. This response can cause local inflammation or, if widespread, lead to a dangerous cytokine storm (Wang et al., 2025a). Previous studies on LPS-challenged mice exposed to elevated temperatures (~39.5 °C) have shown that LPS boosts macrophages to eliminate pathogens and regulate production of pro-inflammatory serum cytokines, such as TNF-α, IL-1, and IL-6, thus controlling the degree of cell damage (Jiang et al., 1999; Ostberg et al., 2000).

Similarly, lipoteichoic acid (LTA) and peptidoglycan, components of Gram-positive bacteria, also exhibit pyrogenic activity. Intraperitoneal administration of LTA to mice led to an increase in body temperature and caused drowsiness (Szentirmai et al., 2021). Lipoteichoic acid (LTA) acts as a major cell surface component of Gram-positive bacteria and has a dual mode of action: it is essential for the bacteria’s physical and physiological functions, but it also triggers host immune responses. In bacteria, LTA helps to regulate ion homeostasis, maintain membrane potential, resist osmotic stress, and contributes to cell wall structure. In the host, LTA can bind to host cells through specific receptors, specifically CD14 and TLR2, or to membrane phospholipids, initiating an immune response that releases inflammatory mediators and can lead to tissue damage and inflammation. This binding triggers immune cells like neutrophils and macrophages to release inflammatory mediators, such as cytokines, reactive oxygen species, and cationic peptides (Ginsburg, 2002). When LTA binds to TLR2 on macrophages, signaling pathways are activated, stimulating the release of IL-1β, IL-6, and TNF-α leading to the development of fever (Guitiérrez Venegas and Cardoso Jiménez, 2006).

Taken together, these examples illustrate that various microbial components such as LPS, LTA, flagellin, CpG-DNA, and bacterial exotoxins can trigger the febrile response through different receptor pathways and cytokine profiles. However, the nature of the elicited immune response, and thus the pattern and intensity of fever, can differ substantially depending on the type of pathogen involved. Understanding these pathogen-specific responses helps to explain why febrile reactions in bacterial and viral infections often display distinct immunological and clinical features. Interestingly, febrile responses exhibit distinct immunological patterns in infected patients that differ significantly depending on the type of pathogen, bacterial or viral. In bacterial infections, an increased expression of C-reactive protein (CRP) has been observed compared to infections caused by viral pathogens (Naess et al., 2014). Furthermore, the concentrations of IL-6 and IL-8 were found to be lower during febrile illnesses of viral origin than during bacterial infections, with IL-8 levels being higher in infections caused by Gram-negative bacteria. During bacterial infections, an elevated platelet count in plasma has also been observed compared to patients infected with virus (Liu et al., 2016). This phenomenon may be attributed to the role of thrombocytes as the first line of defense, recognizing endothelial cell damage during infection. In infections caused by Gram-negative bacteria, lipopolysaccharide can activate platelets, leading to reactive thrombocytosis and, consequently, an increased platelet count. In contrast, during viral infections, the platelet count tends to decrease due to enhanced adhesion of thrombocytes to infected endothelial cells and the inhibition of hepatic thrombopoietin (TPO) synthesis, which normally stimulates platelet production in the bone marrow (Fan et al., 2024).

In summary, external pyrogens are key molecules in inducing and initiating the process of infectious fever. Subsequently, their action, or that of other stressors such as mechanical damage, leads to the induction of internal pyrogens, secreted by the host organism. These molecules enable the activation and communication between cells, particularly those belonging to the immune and nervous systems, responsible for inducing inflammation and elevating body temperature.

## 4 The mechanisms of human body defense under fever

### 4.1 Immune cells activation

One of the most important roles of fever is to raise the body’s temperature to inhibit pathogen growth and to boost immune cell function enabling faster and more effective response against infection. Therefore, it strengthens defense mechanisms against pathogens. In the beginning, immune cells such as macrophages, dendritic cells and neutrophils, kill the pathogens via phagocytosis or direct cytotoxic mechanisms. Elevated temperatures stimulate one of the first innate immune response mechanism by boosting the release of neutrophils from the bone marrow driven by Granulocyte Colony-Stimulating Factor (G-CSF), IL-17 and IL-1. Heat-induced production of chemokine CXCL8 (IL-8) accelerates recruitment of neutrophils. Additionally, fever temperatures increase membrane permeability, facilitating neutrophils migration through vessel walls. Altogether, these responses amplify the overall bactericidal activity (Evans et al., 2015).

An important role of dendritic cells was described under their transformation at febrile temperatures close to 40 °C. Several different studies show that dendritic cells do not show differences in their viability and cytokine release, but they exhibit higher maturation rate and a lower phagocytic capacity at 40 °C. Together with macrophages they are crucial cells to fight the infection, when the fever occurs and they are able to modulate their immune functions along the process (Plaza et al., 2016). Macrophages and dendritic cells are a bridge between innate and acquired immunity – they detect pathogens from peripheral tissues and migrate to the lymph nodes, where they activate T lymphocytes (Evans et al., 2015). When lymphocytes enter the lymphoid organs their response to mitogens and proliferation rate are enhanced by febrile temperatures. Various types of T cells with different functions help to recognize pathogens, control inflammation, and kill infected cells. Studies *in vitro* and *in vivo* based on the APCs antigen-driven T-cell activation models have shown that the CD8^+^ T cells treated with heat show greater differentiation towards an effector phenotype, accompanied by increased production of IFNγ, enhancing cytotoxic function. This molecular activation was also leading to increased enrichment of the CD8+T cells in membrane associated cholesterol. Similar heat-induced changes in membrane fluidity occur in CD4^+^ T cells. Both findings confirms enhanced signaling and differentiation of T cells at febrile temperatures (Mace et al., 2012; Zynda et al., 2015). Immune cells, such as neutrophils, macrophages, dendritic cells and lymphocytes are therefore the first cells, which are involved in host’s response to pathogen invasion, particularly to external pyrogens derived from pathogen’s cells. These cells start to secrete immune response related molecules such as cytokines and antibodies, which work against the pathogens. Furthermore, fever fundamentally alters cellular biochemistry, acting as a switch that ramps up the disease-fighting power of the immune cells. Changes in the body’s core temperature directly influence the nutrient use, energy production, and survival mechanisms of immune cells and it is always described by the term immunometabolism. Every 1 °C rise in body temperature speeds up chemical reactions in the body. Because immune cells need to act fast during an infection, higher temperatures can boost the metabolic capacity of specific white blood cells, such as neutrophils, allowing them to fight pathogens more aggressively (Wensveen et al., 2024; Holder et al., 2025; Wilander and Rathmell, 2025).

### 4.2 Human heat shock proteins

Apart from the pro-inflammatory response the human body produces molecules which activate multiple mechanisms protecting against tissue damage and general organs failure. First of all, the human body produces heat shock proteins (HSPs) in response to increased temperatures, protecting its own proteins from stress induced by infection, damage and finally proteolysis. HSPs play a crucial role in the host’s immune response – they contribute to the release of cytokines, mediators of the immune response. For instance HSP70, which is secreted by various living cells to stimulate immune cells like macrophages, and others HSPs, such as HSP90, which enhances immune cell function including T cell trafficking to infection sites. This creates a feedback loop where fever and the heat shock response work together as a host defense mechanism. In addition, they prevent protein misfolding, refold denatured proteins, and degrade irreversibly damaged ones (Hasday and Singh, 2000; Plaza et al., 2016; Chong et al., 2025).

### 4.3 Reactive oxygen species produced in host organism

Reactive oxygen species (ROS) produced by human body play a crucial role in the host immune response during bacterial infections. They are primarily generated by neutrophils and macrophages during the so-called oxidative (respiratory) burst, which constitutes a fundamental mechanism of innate immunity. This process is driven by the NADPH oxidase complex (NOX2), which transfers electrons to molecular oxygen, leading to the formation of superoxide anions within the phagosome containing engulfed pathogens. Subsequent dismutation and secondary reactions give rise to various ROS with potent antimicrobial properties. In addition, mitochondrial pathways also contribute to ROS production, further enhancing the antimicrobial activity of phagocytic cells (Shekhova, 2020). Furthermore, fever increases the formation of reactive oxygen species (ROS) in various types of human tissues, which can promote both signaling and oxidative stress. For example, a study of Gomes et al. (Gomes et al., 2018) showed increased ROS in the liver, brown adipose tissue, and hypothalamus during a fever, but not in the blood. The body’s immune cells, like neutrophils, increase ROS production to fight pathogens and then leading to acute-phase response to infection, which triggers fever development for example by activating pro-inflammatory pathways, such as NF-ĸB. In addition, fever increases the metabolic demand and oxygen consumption of many organs, including the brain and heart. In the brain, ROS can alter the hypothalamic set point for temperature regulation, contributing to fever itself. Increased cellular metabolism leads to higher ROS production, particularly from mitochondria, which contributes to fever development by several processes including induction of oxidative stress, responsible for the damage of DNA, lipids, and proteins. Persistent oxidative stress can lead to cell and tissue damage, especially in the context of prolonged or severe fever and infection. This increase is linked to the body’s immune response to pathogens and can contribute to inflammation and tissue damage if not balanced by antioxidant defenses (Kayesh et al., 2025). In addition, it has been shown that the immune response is supported by cytoprotective proteins such as Cu/Zn superoxide dismutase (SOD) and hemoxygenase-1 (HO-1), which protect cells from oxidative stress by neutralizing reactive oxygen species (ROS) and catabolizing hem into products with anti-inflammatory properties, thereby preventing tissue damage (Hasday and Singh, 2000).

The mechanisms associated with inducing the host immune response and the supporting ones, such as the production of HSPs or ROS, are highly specialized and developed processes that protect the host from the negative effects of fever. For this reason, on the one hand, they provide conditions in which our body fights pathogens, and on the other hand, they protect itself against damage caused by elevated temperature and excessive immune response.

## 5 General mechanism of infectious fever

**Figure 1:**
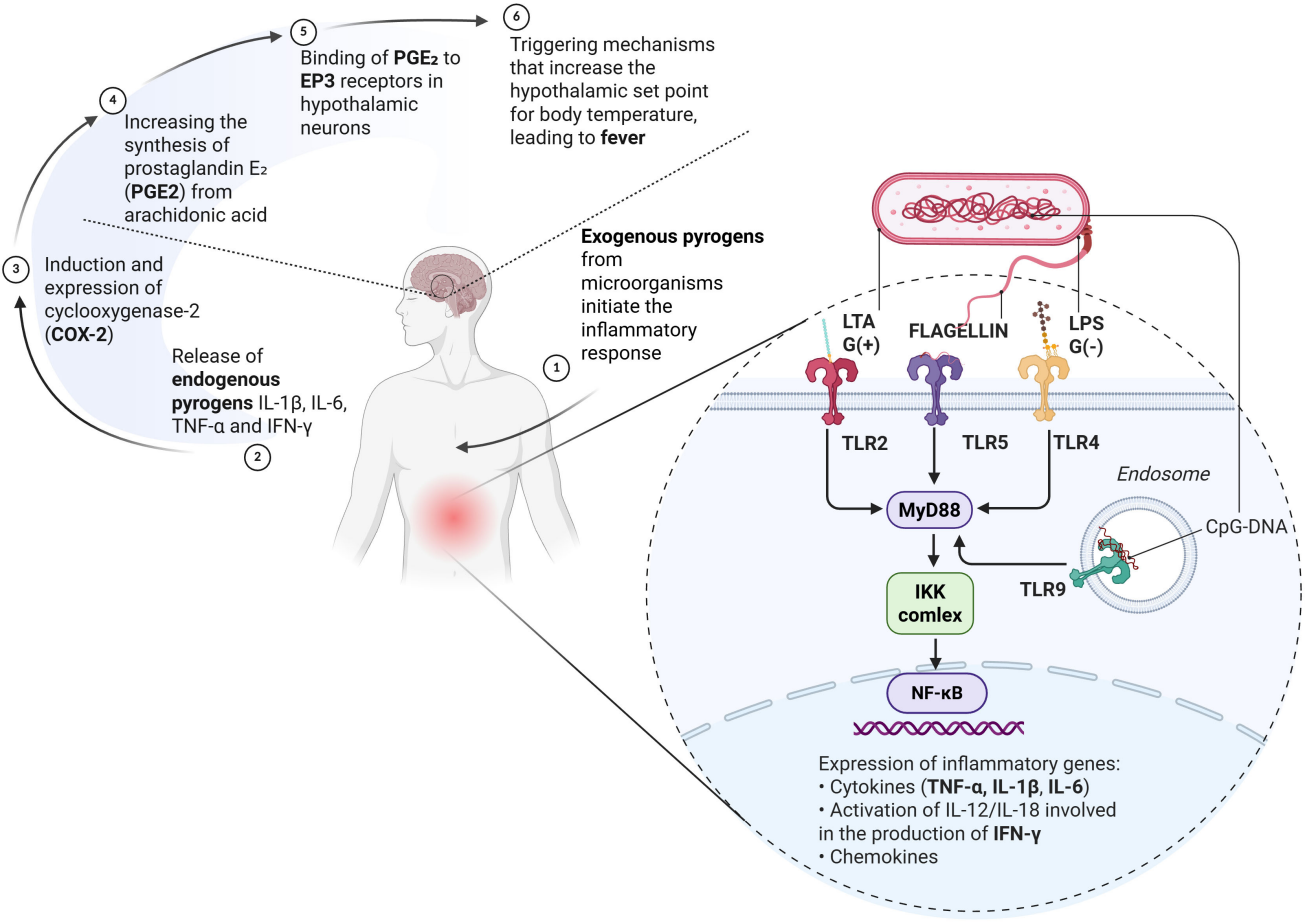
The mechanism of fever development. Exogenous pyrogens derived from microorganisms (e.g., LPS from Gram-negative bacteria and LTA from Gram-positive bacteria) stimulate immune cells to release endogenous pyrogens such as IL-1β, IL-6, TNF-α and, subsequently IFN-γ. These cytokines induce the expression of cyclooxygenase-2 (COX-2) in brain endothelial cells, leading to increased synthesis of prostaglandin E2 (PGE2) from arachidonic acid. PGE2 binds to EP3 receptors on hypothalamic neurons, elevating the temperature set-point and triggering mechanisms that enhance heat production and reduce heat loss. As a result, body temperature gradually rises, leading to the development of fever. Created in BioRender. Kramarczyk, K. (2026) https://BioRender.com/lvwde34.

Infectious fever is induced and maintained through a close interaction between the immune system and the central and peripheral nervous systems. A key mechanism of this process is IL-6-mediated COX-2 induction and subsequent PGE2 production, which links cytokine signaling to the neuronal control of thermoregulation (Li et al., 2026) and it is broadly described (Evans et al., 2015; Li et al., 2026). Briefly, it is associated with the release of endogenous pyrogens, such us interleukin-1-beta (IL-1β), interleukin-6 (IL-6), tumor necrosis factor-alpha (TNF-α) and interferon-gamma (IFN-γ) in a response to exogenous pyrogens (e.g., bacterial and viral antigens). Pyrogenic cytokines act on the central nervous system, particularly on thermoregulatory neurons in the medial preoptic area of the hypothalamus, the main center of thermoregulation (Nilsberth et al., 2009). Their activity leads to the induction and expression of cyclooxygenase-2 (COX-2), a key enzyme in the pathogenesis of fever. COX-2 is the inducible form of the enzyme that participates in the synthesis of prostaglandins from arachidonic acid. Under physiological conditions, its expression level is low or undetectable in most tissues, in contrast to COX-1, which is constitutively expressed throughout the body (Zhang et al., 2003). However, COX-2 expression can be rapidly upregulated in response to inflammatory stimuli or other stress-inducing factors, including changes in body temperature, serving as a protective mechanism in brain endothelial cells. This leads to increased synthesis of prostaglandin E2 (PGE2) from arachidonic acid, which is released from cell membranes during infections. It serves as a precursor for the formation of numerous biologically active compounds that influence the inflammatory response and fever. It can be metabolized through three main enzymatic pathways: the cyclooxygenase (COX) pathway, the lipoxygenase (LOX) pathway, and the cytochrome P-450-dependent epoxygenase (CYP450) pathway. As previously mentioned, the COX pathway leads to the production of prostaglandins, particularly PGE2, which acts in the hypothalamus and is responsible for raising the body temperature. The LOX pathway, in turn, may participate in endogenous antipyresis, the body’s natural mechanism for limiting excessive fever. The least studied, yet increasingly important, is the epoxygenase (CYP450) pathway, which produces compounds such as epoxyeicosatrienoic acids (EETs) and hydroxyeicosatetraenoic acids (HETEs). P-450 enzymes are also present in the brain, including the hypothalamus, suggesting their involvement in temperature regulation. Studies have shown that activation of P-450 enzymes by certain drugs reduces fever and alleviates the inflammatory response, whereas inhibition of these enzymes enhances fever and increases the production of IL-6 and PGE2. These findings indicate that the products of arachidonic acid metabolism play a key role both in the development of fever and its natural limitation, representing an important element in the regulation of the body’s inflammatory response (**Figure 1**) (Kozak et al., 2000). PGE2 binds to EP3 receptors in hypothalamic neurons, triggering autonomic mechanisms responsible for raising the thermal set-point. As a result, the hypothalamus initiates responses that increase heat production and reduce heat loss. A key mediator of these processes is norepinephrine, which enhances thermogenesis in brown adipose tissue and induces vasoconstriction in the skin, limiting heat dissipation. In addition, shivering further generates heat. Consequently, the body’s temperature gradually rises (Evans et al., 2015; Balli et al., 2025).

Fever is the result of a complex interaction between the immune, nervous, and endocrine systems, a process known as neuroendocrine immunomodulation. The endocrine system contributes by releasing antipyretic hormones such as vasopressin (AVP), α-melanocyte-stimulating hormone (α-MSH), and glucocorticoids. These hormones inhibit the effect of cytokines on the hypothalamus, limiting the production of PGE2 and preventing excessive increases in body temperature. In this way, the nervous and endocrine systems work together to maintain a balance between pyrogenic and antipyretic activity (Kozak et al., 2000).

## 6 The role of antipyretics in relieving fever symptoms

Infection primarily induces immune system function, which in turn contributes to the development of fever’s symptoms. Currently used medications, such as aspirin, paracetamol (acetaminophen), and ibuprofen are the most frequently used analgesics, antipyretics and anti-inflammatory medications to relieve pain, fever and/or inflammation. However, their mechanisms of action are slightly different and they have different effects on the human body and immune system. Nevertheless, these drugs not only eliminate unpleasant symptoms, they also weaken the immune response by blocking its specific pathways.

Aspirin reduces inflammation and has a blood-thinning effect. This drug is used to treat pain, but also heart conditions such as heart attack, and blood clotting disorders, including coronary and cerebrovascular thrombosis. It belongs to the group of nonsteroidal anti-inflammatory drugs (NSAIDs) and has anti-inflammatory, analgesic, and antipyretic effects. Aspirin inhibits the activity of the COX enzyme, which leads to the production of prostaglandins, responsible for sensations of pain, fever and inflammation. Aspirin acetylates the hydroxyl of a serine residue in the active site of the COX. The second important mechanism of aspirin’s action is irreversible inhibition of COX, which is involved in the synthesis of thromboxane, a key component in platelet aggregation and blood clotting. This mode of action distinguishes it from other NSAIDs, including diclofenac and ibuprofen, which are reversible COX inhibitors (Fijałkowski et al., 2022). Ibuprofen is an anti-inflammatory drug that also alleviates pain and reduces fever. It is a reversible, non-selective COX inhibitor, which then reduces prostaglandin production, thereby relieving pain, fever, and inflammation in the body (Ainyanbhor et al., 2025). It is still not fully understood how paracetamol works. Similarly to ibuprofen, it is thought to act by blocking the COX enzymes that produce prostaglandins, although through a different mechanism of action. The drug inhibits COX activity only in the central nervous system but does not have anti-inflammatory effect because it cannot bind to COX in the peripheral tissues. This points that paracetamol interacts with the brain’s endocannabinoid system and the descending pain pathway to inhibit pain sensation. Furthermore, paracetamol does not cause gastrointestinal problems or prolong bleeding time, as is the case with aspirin and other salicylates (Graham and Scott, 2005).

The immune system and its specialized immune cells, such as T lymphocytes work more efficiently at higher body temperatures, specifically under fever conditions. The mechanisms of action of fever-reducing agents clearly indicate their modulation of the immune response. Their administration is associated not only with lowering body temperature but also with alleviating pain and reducing the reactivity of the immune system. Thus taking antipyretics to feel better is a good compromise for short-term comfort, but they do not play an essential role in recovery and might slightly slow down the healing process in some infections. NSAIDs exert an inhibitory effect on neutrophils and alter their functions associated with degranulation, phagocytosis, migration and ROS production. For instance, aspirin administration before or immediately after the tracheal instillation of *Streptococcus pneumoniae* in mice model of pulmonary infection was associated with lower bacterial clearance and a higher mortality (Esposito, 1984; Kaplan et al., 1984). It has also been observed that patients with COVID-19 pneumonia who developed a fever above 39 °C have better survival rates than those who did not develop a high fever (Wu et al., 2020). Recent studies also suggest that taking NSAIDs before vaccination against COVID-19 to prevent side effects is generally discouraged, as it might potentially blunt the immune response the vaccine aims to create (Skarke et al., 2023). Therapy based on antipyretic medication and external cooling which included >15–000 ICU patients with sepsis showed no beneficial effect on reducing mortality risk with the use of antipyretic therapy and showed that external cooling may even be harmful for those patients (Zhang et al., 2015). Based on the study of Weinstein et al., a body temperature greater than 38 °C was associated with increased survival in patients with spontaneous bacterial peritonitis. This suggests that within the context of this infection, a febrile response was a positive prognostic factor rather than an adverse one (Weinstein et al., 1978). Therefore, we have to take into account that reducing fever, may slow down important mechanisms of the immune response, which may interrupt healing and interfere with immune clearance of infection.

Antipyretics do not cure the infection itself, but they merely mask symptoms and help combat malaise. A moderately elevated temperature is the body’s natural defense mechanism, which speeds up metabolism, inhibits viral replication, and stimulates the immune system. The indications and potential effects of antipyretics can be divided into two aspects. On the one hand they help by improving the patient’s comfort and well-being, they facilitate sleep, rest, and regeneration or prevent dehydration. In this way, they improve the patient’s comfort and normalize body temperature. An important aspect of using antipyretics for children is, that they protect against febrile seizures. Those drugs are also crucial for immunocompromised patients because they prevent the dangerous metabolic stress of high fevers. Neutropenia patients, who are lacking sufficient white blood cells cannot mount a normal inflammatory response, which means even a minor infection can quickly escalate into septic shock. In these patients, fever is often the primary sign of a life-threatening infection, such as neutropenic sepsis (Strojnik et al., 2016) On the other hand they might be harmful or contraindicated, because they prolong the duration of the illness by blocking the natural defense process, when the fever is low or moderate and they can cause side effects (e.g., liver damage from a paracetamol overdose or stomach irritation from ibuprofen). What makes also an important point, while taking fever reducing drugs, there is always the risk of pathogen transmission to other people, since those drugs mask symptoms, rather than treat infection.

## 7 The harmful effect of febrile temperature on bacteria

Temperature is a key factor affecting most processes occurring in living organisms. Excessively high temperatures affect most of pathogenic microbes by contributing to their protein denaturation, changes in the structure of nucleic acids, and the fluidization of biological membranes. Conversely, excessively low temperatures inactivate proteins, slow down the kinetics of enzymatic reactions, promote secondary structures in nucleic acids, and rigidify membranes disrupting transport and diffusion through membranes (Huus and Ley, 2021). Therefore, temperature changes are highly important not only for the host’s organism, but particularly for the pathogen, which enters the host and have to adapt to new environmental conditions and heat stress associated with fever.

### 7.1 Heat influence on biological macromolecules

The systemic febrile temperature may impair many of the biological processes, which are affected in the pathogens. Among the most crucial housekeeping biomolecules participating in maintaining all living organisms are lipids, nucleic acids and proteins. RNA denatures at approximately 65–70 °C, whereas DNA damage typically occurs between 75 and 90 °C, with the respect that the precise temperature for both nucleic acids depending on the factors such as GC (guanine-cytosine) content and surrounding environment conditions (e.g. pH, salt concentration) (Sadhu et al., 1984). Similarly, lipids exhibit exceptional thermal stability at high temperatures, and it appears that the primary mechanisms, which affect bacteria at higher temperatures corresponding to the onset of fever involve-key proteins in prokaryotic cells, which are mainly affected. They constitute one of the most temperature-sensitive groups of macromolecules and may denature inside bacterial cells during heat shock at hyperthermic temperatures, but can readily unfold and lose their function at lower temperatures, even below the human body temperature range of 37–38 °C (Lepock et al., 1993). This underscores the importance of protein sensitivity under human febrile temperatures.

Studies based on 2384 patients shows that fever in the emergency department strongly predicts lower mortality and improved survival for patients with severe sepsis or septic shock admitted to the Intensive Care Units (ICU). Contrary to traditional belief, higher body temperature at presentation correlates with better outcomes and shorter hospital stays, while low temperature or hypothermia signifies a worse prognosis (Inghammar and Sunden-Cullberg, 2020). Similar conclusion comes from another cohort of 2225 ICU patients which had better survival rate and shorter hospital days when they developed higher fevers. Among overall mortality was 25% more than 50% of patients had a body temperature below 38.3 °C. Mortality was inversely correlated with temperature and decreased for minimum 5% per °C increase, from 50% in patients with the lowest temperatures to 9% in those with the highest body temperature (Sundén-Cullberg et al., 2017). This shows that at the critical stage of the development of sepsis might be supported by the development of fever and increase patients survival, when the standard pharmacological options fail.

One another important factor influencing bacterial cell death under febrile temperature is the surrounding microenvironment and the conditions, which change under elevated temperatures. An important *in vivo* studies carried out in rabbits infected with *Pasteurella multocida* demonstrated that under fever development the concentration of iron in rabbit’s plasma was decreased. This phenomena was confirmed as a defense mechanism against the infection also in *in vitro* studies. *Pasteurella multocida* was able to grow in low and high iron concentration in afebrile conditions. However, while the temperature was increased to febrile temperature the growth of the bacteria was inhibited only in low-iron medium (Kluger and Rothenburg, 1979). Similarly to iron, under febrile conditions an increased metabolic rate and the activation of immune system there is lack of other important ions, such as zinc and cooper, which are necessary for bacterial growth (Stefanache et al., 2023). This observation shows also that there are probably many different cooperating events, which are responsible for bacterial death in febrile temperatures. These studies, carried *in vitro* have some limitation comparing to *in vivo* studies, however allow for studying the exact influence of important ions on bacterial survival under febrile stress. Similarly, studies on *Salmonella* strains have shown that the bacteria is also more viable to surrounding bactericidal factors *in vitro*, such as NaCl or glycerol while is exposed to 37 °C, than when is cultured at 21 °C (Mattick et al., 2000).

**Figure 2:**
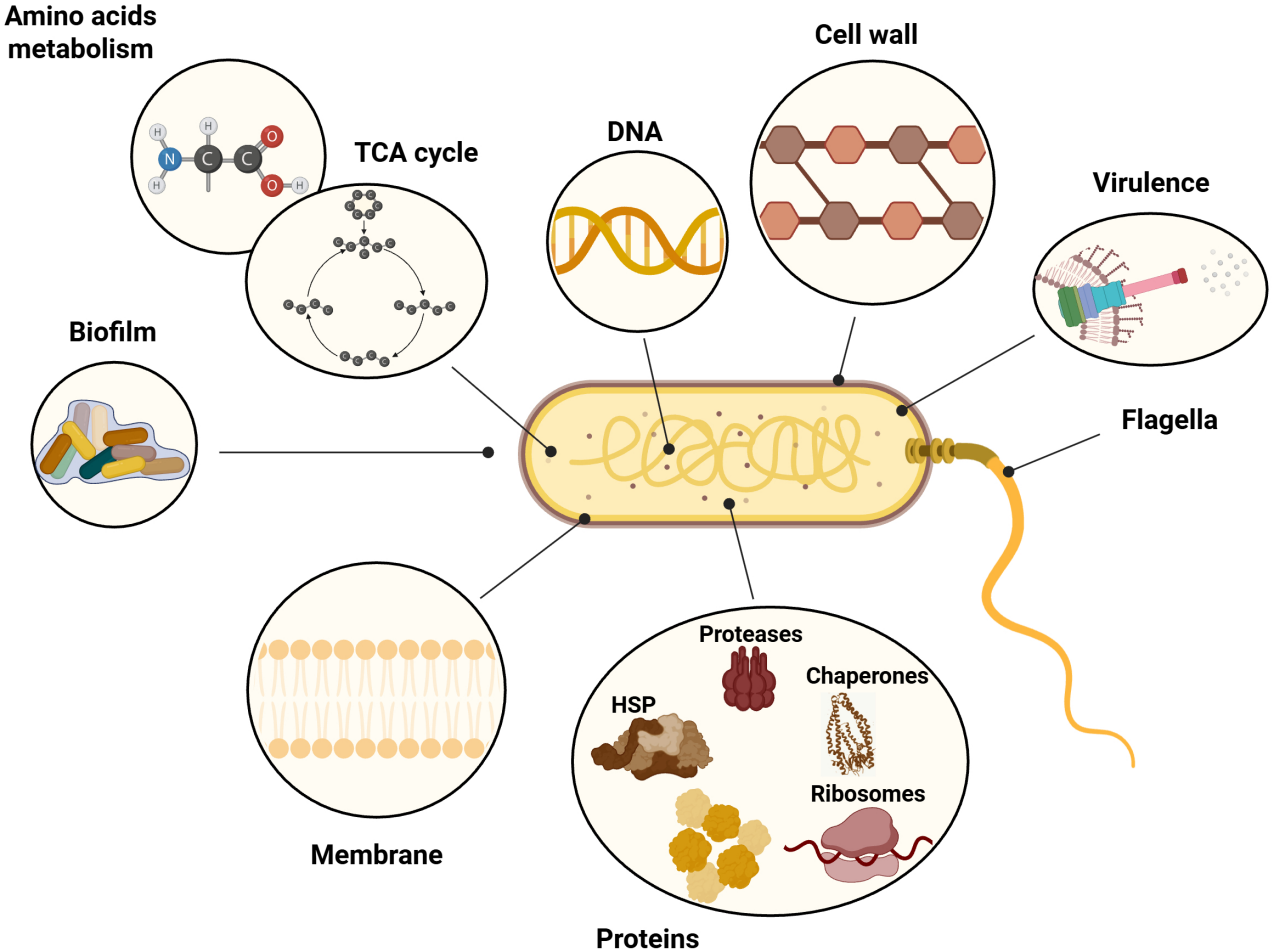
The bacterial cell components, which are affected under febrile temperatures. Critical areas within the bacterial cell where homeostatic mechanisms are disrupted and modulated under elevated temperatures. Chaperone structure adapted from Korndörfer et al (Korndörfer et al., 2004). Created in BioRender. Kramarczyk, K. (2026) https://BioRender.com/5g07p9h.

The primary and most immediate threat of thermal stress, particularly a sudden heat shock, is proteotoxicity. Elevated temperatures provide sufficient energy to break the non-covalent bonds that maintain a protein’s precise three-dimensional structure, causing essential enzymes and structural proteins to unfold (Fleury et al., 2009; Sun, 2017; Yan et al., 2024). This exposure to elevated temperatures triggers significant physiological changes in bacteria. The cell’s first response involves the rapid and tightly regulated induction of heat-shock genes (HSGs), which encode heat-shock proteins. This response is managed by a conserved, multi-layered network of protein quality control systems (Fleury et al., 2009; Chen et al., 2011; Yan et al., 2024). Since proteins lose their function and stability, this cytoprotection relies on HSPs, which include molecular chaperones and proteases (Craig and Schlesinger, 1985). The system is designed to sort damaged proteins into two classes: salvageable, which are refolded by chaperones, and terminally damaged proteins, which are destroyed by proteases (Fleury et al., 2009; Chen et al., 2011; Yan et al., 2024). Analysis across diverse bacterial species reveals that the core protein quality control systems response is universally conserved but exhibits advanced, time-dependent regulation (Fleury et al., 2009; Yan et al., 2024). Regulation of heat shock genes and proteins in bacteria has been comprehensively reviewed in many studies (Rosen and Ron, 2002; Maleki et al., 2016; Schumann, 2016; Roncarati and Scarlato, 2017; Chinedu, 2023). The proteomic analysis revealed the significant differences in the expression of several protein groups, including: cell envelope proteins, enzymes involved in oxidative stress, central metabolism and amino acid biosynthesis, chaperons and other proteins involved in protein synthesis (**Figure 2**) (Lüders et al., 2009).

### 7.2 Fever influence on the outer membrane proteins

Heat mediated remodeling involved a shutdown of normal growth processes, affected by the down-regulation of key proteins in *E. coli*. In *in vitro* studies the outer membrane protein A (OmpA), which stabilizes the outer membrane and maintains the cell’s rod shape, and L-fucosamine synthetase (FnlC), an enzyme involved in building the O-antigen to the LPS, were produced in 42 times and 20.5 times lower amounts, respectively. In contrast, the cell also mounted an active stress response. The outer membrane porins OmpC and OmpF were 30-fold up-regulated (Lüders et al., 2009). These two proteins are regulated by temperature and osmolarity (Lugtenberg et al., 1976; Nikaido and Vaara, 1985) and a higher number of these porins increases the cell’s capacity for nutrient assimilation, which is needed to sustain metabolism (Özkanca and Flint, 2002; Lüders et al., 2009). To manage this massive influx of new outer membrane proteins (OMPs), the Skp chaperone, which ensures their correct folding, also showed a 34-fold increase in *E. coli* (Lüders et al., 2009).

### 7.3 Membrane fluidity and lipid metabolism in bacterial thermoadaptation

Lipid membranes act as semi-permeable barriers that support essential cellular processes, including cell wall construction and substance transport. These functions critically depend on membrane fluidity, which is highly sensitive to temperature and tightly regulated by the composition of the phospholipid bilayer, particularly the balance between saturated and unsaturated fatty acid chains. At low temperatures, membranes become more compact and less permeable, whereas at elevated temperatures they become excessively fluid. To maintain optimal membrane functionality, bacteria actively adjust lipid composition (Harayama and Riezman, 2018). One of the primary adaptive strategies involves remodeling membrane fatty acids. At lower temperatures, bacteria increase the proportion of unsaturated fatty acids (UFAs), whose double bonds introduce structural kinks that prevent tight lipid packing and enhance fluidity. Conversely, at higher temperatures, cells enrich saturated fatty acids (SFAs), which allow tighter packing of acyl chains, thereby reducing membrane fluidity and increasing rigidity (Harayama and Riezman, 2018; An et al., 2023; Wang et al., 2023a). This shift creates a more stable membrane that is less susceptible to thermal disruption. A clear example of this adaptation is observed in *Enterococcus faecium* RS047, where thermotolerant strains in *in vitro* studies increase total SFA content from 44.14% to 49.29%, primarily through elevated levels of C16:0 (hexadecanoic acid) and C18:0 (octadecanoic acid). Simultaneously, the proportion of UFAs decreases from 55.85% to 50.71%, largely due to reduced oleic acid (C18:1) (Wang et al., 2023a). These compositional changes enhance membrane rigidity and stability under heat stress (An et al., 2023; Wang et al., 2023a).

Bacteria also regulate membrane fluidity through metabolic and genetic control mechanisms. In *Escherichia coli*, membrane composition is adjusted indirectly by modulating the availability of fatty acid precursors used in phospholipid synthesis. Key enzymes such as PlsB, which incorporates both saturated and unsaturated fatty acids, and PlsC, which preferentially incorporates unsaturated fatty acids, play central roles, with PlsB acting as a major determinant of membrane fluidity. Additionally, two opposing transcription factors, FadR and FabR, regulate fatty acid biosynthesis: FadR activates the *fabA* and *fabB* genes to promote UFA synthesis and repress degradation, while FabR inhibits these genes to prevent excessive membrane fluidity at higher temperatures (Fujita et al., 2007; Hoogerland et al., 2024). Unlike some organisms, *E. coli* does not directly sense membrane fluidity. In contrast, *Bacillus subtilis* employs a dedicated sensing system, the DesK–DesR two-component regulatory system. When temperatures fall below approximately 30 °C, DesK detects membrane rigidification and becomes active as a kinase, phosphorylating the response regulator DesR. Activated DesR induces expression of the *des* gene, encoding a Δ5-lipid desaturase that introduces double bonds into fatty acids, thereby increasing membrane fluidity under cold conditions. When unsaturated fatty acids accumulate excessively, feedback mechanisms suppress *des* expression to restore balance (Aguilar et al., 2001). Beyond compositional remodeling, thermotolerance strategies also involve broader metabolic reprogramming and protection of lipids from oxidative damage (Fleury et al., 2009; Lüders et al., 2009; An et al., 2023; Wang et al., 2023a). For instance, bacteria may reduce the synthesis of branched-chain fatty acids, which disrupt orderly lipid packing, and shift toward longer-chain fatty acids, further contributing to membrane stabilization at elevated temperatures (Wang et al., 2023b).

### 7.4 ROS effect on pathogens

At the molecular level, ROS exhibit broad cytotoxic effects by targeting nearly all classes of biomolecules within microbial cells. One of the key mechanisms involves oxidative damage to DNA, including base modifications, the formation of abasic (AP) sites, and single-strand breaks (SSBs). When such lesions occur in close proximity, they may result in double-strand breaks (DSBs), which are among the most cytotoxic forms of DNA damage. While unrepaired or improperly repaired DSBs often lead to cell death, oxidized base lesions are primarily associated with mutagenesis and genomic instability (Hegde et al., 2012).

Simultaneously, ROS induce lipid peroxidation in microbial cell membranes, disrupting their integrity and fluidity. This results in damage to both the cell wall and the cytoplasmic membrane, increasing membrane permeability and leading to leakage of intracellular components such as nucleic acids and cytoplasmic contents. Consequently, cellular homeostasis is lost, ultimately resulting in cell death (Liu et al., 2024). In addition to lipids and DNA, ROS also target the cellular proteome. Oxidative modifications of proteins, such as carbonylation, lead to protein misfolding, enzyme inactivation, and disruption of essential metabolic pathways. Because these effects occur simultaneously across multiple cellular systems, ROS-based antimicrobial mechanisms are considered less prone to the development of resistance compared to conventional antibiotics (Vaishampayan and Grohmann, 2021).

Despite their potent antimicrobial activity, bacteria have evolved a variety of adaptive strategies to counteract ROS-mediated damage. One such strategy involves the upregulation of ROS-scavenging systems, which is widely conserved across bacterial species (Periago et al., 2002; Fleury et al., 2009; Lüders et al., 2009; Wang et al., 2023a). Reactive oxygen species, including superoxide (O_2_^-^) and hydrogen peroxide (H_2_O_2_), can cause severe oxidative damage, particularly through lipid peroxidation. To mitigate these effects, bacteria such as *Escherichia coli* increase the production of specific protective proteins. Superoxide dismutase (SodA) catalyzes the conversion of superoxide into hydrogen peroxide, which is subsequently detoxified into water by catalases or alkyl hydroperoxide reductase (AhpC). Both SodA and AhpC showed a 17.7-fold increase in abundance in *E. coli* under elevated temperature conditions (Lüders et al., 2009).

Additionally, *E. coli* upregulates the DNA-binding protein Dps, which compacts genomic DNA into a nucleoid structure that is less susceptible to oxidative stress, with its levels increasing 7.3-fold (Lüders et al., 2009). Similar adaptive responses have been observed in other bacteria, including *Bacillus cereus*, which induces SodA and TrxA under heat stress (Periago et al., 2002), and *Staphylococcus aureus*, where sodA and trxA genes are upregulated at elevated temperatures (Fleury et al., 2009). These coordinated responses highlight the evolutionary importance of ROS detoxification systems in bacterial survival under hostile environmental and host-imposed conditions (**Table 1**).

**Table 1:** Membrane remodeling and ROS defense strategies.

Defense strategy	Bacterium	Molecular mechanism	Functional outcome
Membrane Remodeling (Lipid Profile)	*Enterococcus faecium* RS047	↑ Saturated Fatty Acids (SFA) (C16:0, C18:0)	Tighter packing of acyl chains creates a stable barrier resistant to thermal disruption (Wang et al., 2023a)
		↓ Unsaturated Fatty Acids (UFA) (C18:1)	
		↓ Branched-chain FAs	
ROS Scavenging (Enzymatic)	*Escherichia coli*	↑ SodA and AhpC	Converts superoxide (O_2_^-^) to hydrogen peroxide (H_2_O_2_), then to water (H_2_O), preventing lipid peroxidation (Lüders et al., 2009)
	*Bacillus cereus*	↑ SodA and TrxA	Conserved response to neutralize ROS generated by heat stress (Periago et al., 2002)
DNA protection	*Escherichia coli*	↑ Dps (DNA-binding protein from starved cells)	Transforms genomic DNA into a compact, resistant form less sensitive to ROS and heat (Lüders et al., 2009)

Crucial strategies, that bacteria use to protect their most vulnerable components, specifically the cell membrane and DNA, from heat-induced damage and the resultant oxidative stress.

In the context of current therapeutic challenges, reactive oxygen species represent a promising tool in combating biofilms and multidrug-resistant (MDR) pathogens. Their nonspecific oxidative activity enables them to bypass classical mechanisms of bacterial resistance, making them an attractive alternative or adjunct to traditional treatments. However, their potential cytotoxicity toward host cells necessitates precise control and targeted delivery in therapeutic applications to minimize adverse effects and ensure treatment safety (Alfei et al., 2024).

### 7.5 Bacterial heat shock proteins

It has been noticed that denatured proteins interact with each other by aggregating but also with other native proteins causing their destabilization. This is why it is important to activate the expression of heat shock proteins in response to heat stress which have a protective function - they stabilize and repair denatured proteins or direct them to degradation, thus protecting the cell from damage (Mogk et al., 2018). As mentioned earlier, an elevated temperature in the host body also stimulates pathogens to produce HSPs. Studies have shown that some of them, *Legionella pneumophila* Hsp60, *Escherichia coli* GroEL, *Mycobacterium tuberculosis* Hsp70, M*ycobacterium leprae* HSP65, and *Mycobacterium bovis* Hsp65, can strongly activate host’s innate defense mechanisms. Bacterial HSPs usually induce the expression of several pro-inflammatory factors in host organism such as interleukins, specifically IL-1α, IL-1β, IL-6, tumor necrosis factor TNF-α, and granulocyte–macrophage colony-stimulating factor mRNA (Retzlaff et al., 1994).

One of the most important post-translational modifications of proteins which can also be induced in response to heat shock is phosphorylation. Several bacteria-based studies have shown that DnaK – main chaperone protein HSP70, is autophosphorylated during heat stress, which increases its affinity to substrates, for example in the case of DnaK, higher affinity to unfolded proteins is observed (Moon et al., 2023). Heat of fever modulates the expression of virulence genes and synthesis of specific proteins, enhancing bacterial expansion within the host. In *Staphylococcus aureus*, elevated temperatures up-regulate expression of the *agr* operon and *hld*, *psmα/β* genes, compared to lower ones. As a result, bacteria produce more toxins, such as hemolysins and staphyloxanthin, but form less biofilm which makes them more virulent. Literature shows that increase in host temperature also modifies regulation of virulence genes expression in several other bacteria, e.g. *Salmonella*, *Yersinia* and pathogenic *E.coli*, with an enhancement of regulation observed at temperatures around 42 °C compared to 37 °C (Brewer et al., 2021; Huus and Ley, 2021). In *Pseudomonas aeruginosa*, higher temperature around 39 °C, increases the production of enzymes such as lipases and proteases, as well as biofilm formation, which facilitates its survival and tissue damage (Solar Venero et al., 2024). It has been shown that temperature changes up to 42 °C do not significantly affect the levels of ribosomal proteins and RNA (Morgan et al., 2018). Temperatures elevated to around 42 °C even contribute to increased translation rate. However, the main challenge is correct protein folding which is not adapted to high temperatures. To facilitate the proper assembly of newly synthesized polypeptide chains, bacteria activate molecular chaperones (Moon et al., 2023). At the host’s standard body temperature, bacterial proteins maintain their stability. An increase in temperature beyond normal can lead to protein denaturation contributing to cell death. To counteract this, under unfavorable conditions bacteria activate chaperone proteins, such as ClpB, DnaK, IbpA/B, which prevent protein aggregation, repair damaged proteins and promote bacterial virulence (Lee et al., 2016).

During heat stress denatured, misfolded or damaged proteins accumulate, which can be toxic to the cell. To counteract this, bacteria secrete several various proteases that digest proteins into smaller harmless peptide fragments. In bacteria, several proteases are induced by heat (Lon, ClpP, HslUV, FtsH). They consist of two domains – ATPase and protease domain, that together form a barrel-shaped complex, which encloses substrate proteins targeted for degradation. Upon recognition of the substrate, proteases unfold the substrate protein using energy from ATP hydrolysis. Then, when the protein is unfolded, the polypeptide chain is translocated to the proteolytic chamber where the actual degradation takes place, i.e. proteases degrade proteins into smaller peptide fragments without consuming ATP (Moon et al., 2023).

Bacterial HSPs are highly conserved molecular chaperones that play a critical role in bacterial survival and adaptability. Their primary role is to assist in proper protein folding, stabilize proteins under environmental stress, and prevent harmful protein misfolding or aggregation. Therefore, they are important macromolecules, which support bacteria against the consequences of infectious fever occurring in host organism.

## 8 Bacterial temperature sensing

**Figure 3:**
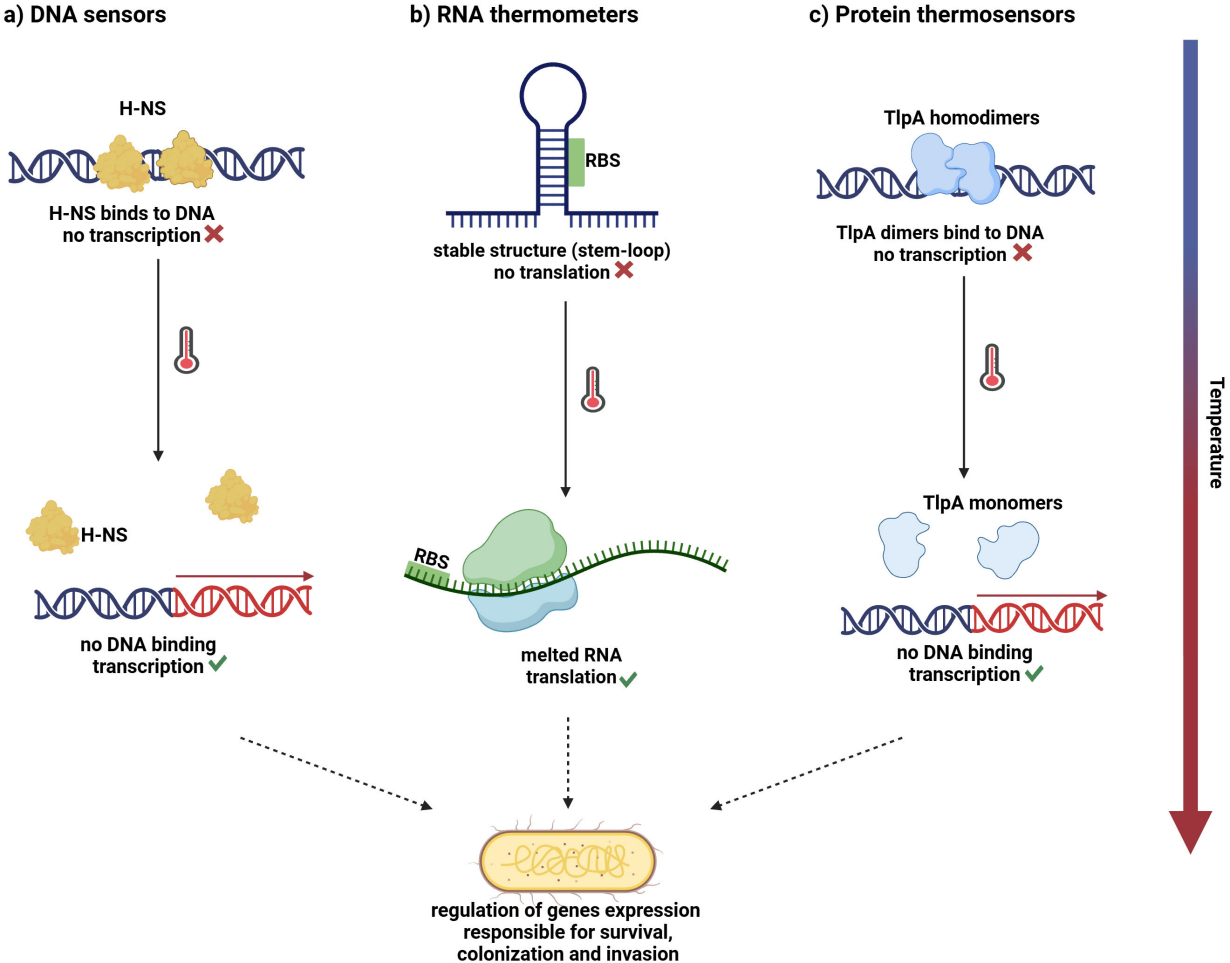
Bacterial temperature sensors enabling adaptation to the environment. **(a)** At low temperatures, H-NS binds to DNA, inhibiting gene expression. At higher temperatures, H-NS loses its ability to bind to DNA, enabling bacterial gene expression. **(b)** At low temperatures, RNAT forms a stem-loop that covers the ribosome binding site (RBS) and prevents bacterial gene translation. Elevated temperatures leads to RNAT melting, exposing the RBS and allowing gene translation. **(c)** At low temperatures, TlpA forms homodimers, which allow it to bind DNA and inhibit bacterial gene transcription. At higher temperatures, the dimers dissociate into monomers, and TlpA loses its ability to bind DNA, activating gene transcription. Created in BioRender. Kramarczyk, K. (2026) https://BioRender.com/1g9gbpi.

Bacterial temperature sensing is an ability of bacteria to detect and respond to fluctuations in temperature. Temperature shifts often regulate expression of genes responsible for bacterial virulence and invasion, allowing them to adapt their metabolism from environmental conditions to those existing in the host organism, which facilitates survival and colonization (Steinmann and Dersch, 2013). There are several temperature sensors, but they can be divided into three main groups - sensors based on DNA, RNA or proteins (**Figure 3**) (Roncarati et al., 2025).

### 8.1 DNA sensors

At the DNA level, in many bacterial species, the most crucial role plays DNA supercoiling which decreases with heating, allowing easier access for transcriptional machinery, so that bacteria can respond more quickly to changing environmental conditions and adapt more easily to host conditions. DNA thermosensors operate by changing the topology and structure of DNA, which in turn influences gene expression. Temperature changes alter the degree of DNA supercoiling, which affects the accessibility of the promoter to RNA polymerase and thus regulates transcription (Roncarati et al., 2025).

In *Salmonella*, elevated temperatures induce positive DNA supercoiling, which activates the expression of the SPI-1 gene, a key determinant of bacterial pathogenicity (Dawoud et al., 2017). Another important DNA temperature sensor is a Histone-like Nucleoid Structuring protein (H-NS). Changes in DNA structure affect the H-NS binding. At low temperatures, H-NS binds to DNA, repressing gene expression. As temperature increases, H-NS loses its ability to bind to DNA, leading to derepression of these genes and allowing their expression in the host (**Figure 3A**) (Roncarati et al., 2025). Important factors involved in H-NS regulation include, for example, regulation of SPI-1 gene activation in *Salmonella enterica* or hemolysin expression in pathogenic *E. coli*, enabling bacterial cells to combat heat stress or ensure successful infection (Ono et al., 2005; Roncarati et al., 2025). Recently, it was discovered that *Yersinia enterocolitica* can respond to temperature changes through DNA methylation. *Y. enterocolitica* has multiple DNA adenine methylase (Dam) sites but only some of them have been shown to be differentially methylated at temperatures of 22 and 37 °C, which influences gene activity. This methylation occurs in promoter regions, as well as in transcription regulators such as LysR and PadR, and genes involved in c-di-GMP metabolism. This may be how *Y. enterocolitica* uses DNA methylation as an epigenetic mechanism to regulate changes in environmental temperature (Van Hofwegen et al., 2023).

### 8.2 RNA thermometers

There are bacteria that activate virulence and stress genes only at temperatures close to the host’s body temperature and not at lower ones. Such regulation was not controlled by proteins, but changes in the secondary structures of mRNA. One of the well-known temperature sensing mechanisms are the RNA thermometers (RNATs). RNATs are located in the 5’ untranslated region (UTR) of mRNA and act as temperature sensors, affecting protein translation. The mechanism of action of RNATs involves changes in their secondary structures in order to adapt to temperature shifts. At low temperatures, below 30 °C, RNATs form stable secondary structure, for example stem-loop, which covers the ribosome binding site (RBS), preventing translation of the gene below. When temperature rises to the host’s body temperature, heat energy melts RNAT structure and exposes ribosome binding site, allowing translation of the gene (**Figure 3B**) (Loh et al., 2018). In *E. coli*, elevated temperatures associated with fever affect mRNA, changing its structure and enabling synthesis of heat shock proteins (Miwa and Taguchi, 2025). One of the first described RNAT was discovered in *E. coli* where *rpoH* gene was a regulatory factor in response to heat shock. At low temperatures it forms a stable secondary structure to block the translation, and destabilizes at higher ones, allowing the process to begin (Morita et al., 1999). Certain bacteria, e.g. *Listeria monocytogenes*, can use RNA thermometers to activate virulence genes. The expression of these genes is regulated by *prfA* whose activity is regulated by temperature. Maximum expression of virulence genes is achieved at 37 °C enabling bacteria to infect the host cells, while at 30 °C their expression is almost blocked. For instance, *Listeria monocytogenes* is able to use temperature as a signal to activate the certain genes needed for infection (Johansson et al., 2002). Higher temperature is also a signal for *Neisseria meningitidis*, which uses RNA thermosensors to avoid host immune response. In response to elevated temperatures, RNA thermosensors activate the translation of factor H binding protein (fHbp). As a result, fHbp binds to human factor H, which inhibits the complement system, allowing the bacteria to avoid lysis by complement (Loh et al., 2013).

### 8.3 Bacterial protein thermosensors

Proteins perform many key functions in living organisms but are highly sensitive to environmental factors, particularly temperature fluctuations. Changes in temperature can alter protein conformation, thereby modulating their stability and activity. Protein thermosensors are molecules that change their structure or conformation in response to temperature shifts. They regulate transcription, enzymatic activity, and signaling, unlike RNA thermal sensors, which operate at the translational level. There are several mechanisms by which proteins can respond to temperature changes, including partial denaturation, alterations in oligomerization and conformational states, and modifications in the phosphorylation status of proteins or membrane components. Partial denaturation leads to the loss of the specific protein function, whereas temperature-induced changes in oligomeric complexes can alter their conformation or cause dissociation into subunits, resulting in modified or lost activity. Additionally, some proteins switch their enzymatic activity in response to temperature changes, for instance, through changes in their phosphorylation state or that of associated membrane proteins (Klinkert and Narberhaus, 2009; Steinmann and Dersch, 2013).The TlpA protein from *Salmonella enterica* serovar Typhimurium is one of the best-characterized protein thermosensors. It acts as a transcriptional repressor that forms homodimers at low temperatures, allowing it to bind DNA and inhibit gene transcription. As the temperature rises, conformational changes weaken the dimeric interactions, leading to dissociation into monomers. As a result, TlpA loses its DNA-binding ability, leading to activation of the target genes (**Figure 3C**) (Klinkert and Narberhaus, 2009). Such temperature-dependent regulation of specific genes at the host’s body temperature increases the virulence of *Salmonella* by activating expression of virulence-related factors. Another example of a temperature-regulated protein sensor is the RovA, found in pathogenic *Yersinia* species. RovA is a transcriptional activator that controls the expression of numerous genes involved in metabolism, stress response, and bacterial invasiveness, for instance, it activates *invA* gene which encodes invasin – a protein responsible for host colonization. Like most thermosensors, RovA protein is sensitive to temperature fluctuations, allowing bacteria to adapt to environmental conditions. At lower temperatures about 25 °C, that protein forms a stable conformation that enables it to bind DNA and activate virulence genes, promoting invasion. However, at the host’s body temperature (37 °C), RovA undergoes partial unfolding, which prevents DNA binding and leads to repression of virulence gene expression. Moreover, RovA becomes unstable and is degraded by Lon proteases at 37 °C. Importantly, these temperature-induced conformational changes are reversible – when bacteria return to 25 °C, RovA refolds and restores its ability to bind DNA and regulate gene expression (Herbst et al., 2009; Quade et al., 2012). Furthermore, the expression of heat shock protein (HSP) genes is controlled by temperature-sensitive transcriptional regulators, such as HrcA, CtsR, and RheA, which protect the microbial cell from heat stress (Roncarati et al., 2025).

For many bacteria, a temperature of 37 °C serves as a signal that they are in the host organism, which activates the defense mechanisms, triggering the modulation of gene expression and usually activates those responsible for survival, virulence and invasion, while repressing others (Plaza et al., 2016). At temperatures around 39 °C, *Staphylococcus aureus* shows increased expression of genes involved in virulence and survival, which in turn contributes to elevated IL-8 level in the host, indicating the activation of defense reactions (Solar Venero et al., 2024). *Salmonella* can sense elevated temperatures in the host organism and enter a persistent state, slowing its metabolism and becoming less visible to macrophages. They slow down the motility and epithelial cell invasion. This occurs because *Salmonella* silences expression of genes in the type III secretion system, which allows bacteria to inject their components into and penetrate host cells, as well as those encoding flagellum and chemotaxis - genes necessary for movement and host cell discovery. On the other hand, *Salmonella* activates some genes called pathogenicity island 2 genes, which enable bacteria to survive infection within cells. However, this strategy allows the bacteria to quickly resume infection once temperatures return to normal (Elhadad et al., 2015).

Processes involved in bacterial temperature sensing allow the bacterium to adapt into changing environmental conditions and they play a role for the pathogen survival under fever development, being a part of an important host-pathogen interaction process.

## 9 Modulation of critical bacterial processes under febrile temperatures

### 9.1 Growth rate and replication

The impact of temperature on bacteria growth and survivability is not always straightforward and may be affected by changing temperatures and the surrounding environmental conditions. Among many different species of bacteria, *Escherichia coli* is one of the most frequently used model organisms that appears to grow optimally at temperature around 37 °C (Shenhar et al., 2009; Solar Venero et al., 2024). *In vitro* studies show that with an increase in temperature, the growth rate goes slightly up till around 40 °C. Then a rapid decline of growth rate is observed up to 25% of maximum growth inhibition at 47 °C (Yang et al., 2020). Other species of bacteria such as *Staphylococcus aureus* exhibit increased growth rates and survivability in arginine and proline deficient media at fever-like temperatures (above 37 °C). Based on the microarray and RNAseq analysis, researchers found upregulated genes from the tricarboxylic acid (TCA) cycle (*gltA*, *acnA*, *icd*, etc.). In addition to that, genes related to arginine are also upregulated. Those alterations appear to increase the rate of growth even in arginine lacking environments (Solar Venero et al., 2024).

Multispecies bacterial colonies exhibit a more complex relationship with temperature that cannot be explained by a straightforward comparison to a logistic model (Knapp and Huang, 2022). This complexity is attributable to species-specific optimal growth temperatures and interspecies interactions, which can alter competitive dynamics under thermal stress (Lax et al., 2020). Transcriptomic analysis using RNA-seq on bacterial co-cultures, specifically of *Pseudomonas aeruginosa* and *Staphylococcus aureus*, compared to monocultures, demonstrates differential adaptation to heat within a 3 °C range, from 37 °C to 40 °C. They observed distinct gene expression profiles in both bacteria, with fold changes >1.5 in genes encoding cell surface proteins and secretion factors, including toxins and exoenzymes. In addition to RNA-seq analysis, assessments of biofilm formation and motility rates indicated that co-cultures of *S. aureus* and *P. aeruginosa* exhibit enhanced survivability but reduced motility under *in vitro* conditions (Solar Venero et al., 2024). .

### 9.2 Gene expression

Among many levels of adaptation, gene expression and its regulation play a crucial role under thermal stress. In the context of genome-wide upregulation of gene expression, pathways associated with survival are frequently enhanced, along with the activation of specific virulence-related pathways (Thomas and Wigneshweraraj, 2014; Waldvogel and Pfenninger, 2021). Virulence factors such as hemolysins, which induce lysis of erythrocytes are often up-regulated under fever-like conditions, this particular regulation is an essential source of hem influencing the survivability of pathogens (Lam et al., 2014). This upregulation is often mediated by complex thermoregulation strategies, many of which are based on RNA thermometers. For instance, in *Listeria monocytogenes*, temperature variations influence the translational efficiency of PrfA, while in *Neisseria meningitidis*, genes like CssA, FHbp, and Lst are similarly affected (Roncarati et al., 2025). *In vitro* analysis of hemolytic activity in *Staphylococcus aureus* shows it getting higher with increasing temperatures within fever like range from 37 °C to 39 °C (Solar Venero et al., 2024). There are many bacterial species, whose gene regulation in a response to heat stress was broadly described [**Table 2**]. Many of those regulations seem to work on multi-level control integrating changes within DNA topology, RNA structuring and protein conformation and activity (Lam et al., 2014; Thomas and Wigneshweraraj, 2014; Roncarati et al., 2025).

**Table 2:** Temperature impact on bacterial genes regulation.

Bacteria	Genes	Related functions	Temperatures
*Klebsiella pneumoniae* ST307 (Müller et al., 2024)	Upregulated: *ibpAB*, *fur*, *sodA*, *acrAB*, *mrkABCD*	Adhesion/biofilm Heatshock response Reduce oxidative stress	37 °C -> 40 °C/42 °C
	Downregulated: *fimHGFDCIA*, *rfb*	Surface attachment LPS biosyntesis	
*Staphylococcus aureus* (Bastock et al., 2021)	Upregulated: *dps*, psm*a*	DNA protection, Virulence factors	34 °C-> 37 °C/40 °C
	Downregulated: *sak*, *sbi*, *agrD* with significant changes with few sRNA	Immune response evasion	
*Vibrio cholerae* (Weber et al., 2014)	Upregulated: *ctxB* at 37 °C, *toxT*	Virulence factors	25 °C -> 30 °C/37 °C/42 °C
*Streptococcus aureus* (Smoot et al., 2001)	Upregulated: *spy0167*, hemolysins gene	Immune response evasion	29 °C -> 37 °C/40 °C
*Pseudomonas aeruginosa* (Cornforth et al., 2018)	Upregulated: *faoA*, *faoB*	Fatty acid oxidation	37 °C -> human infection*
	Downregulated: Quorum sensing related genes	–	
*Leptospira interrogans* (Lo et al., 2006)	Upregulated: AcrR and MarR family	Antibiotic resistance	30 °C -> 37 °C/39 °C
	Downregulated: Cobalamin syntesis	–	

*human infection meaning comparison between bacteria cultivated in laboratory conditions and bacteria taken from human infected than cultivated within 37 °C.

Examples of up- and down-regulated bacterial genes, across different ranges of temperature in which the bacteria were cultivated.

Beyond these regulatory mechanisms, bacterial adaptation to fever temperatures also encompasses a remarkable capacity for genetic instability, manifested through increased mutation rates that can accelerate evolutionary responses to thermal challenges, over temperatures ranging from 21 °C to 41 °C, with significant changes occurring within fever-like temperatures (37 °C–41 °C). However, these changes do not proceed linearly, as temperatures rising above the bacteria’s optimal growth conditions induce stress (Chu et al., 2020). This phenomenon is often related to bacteria mutation towards changes in bacteria resistance to various antibiotics. Study by Eldijk et al (Van Eldijk et al., 2024). working on *E. coli* shows increased mutation rate toward rifampicin resistance but decreased mutation rate towards ampicillin resistance. Those studies were based on sequencing the known loci for resistance genes in samples from bacteria cultured in temperatures 37 °C and 40 °C (Trindade et al., 2012; Van Eldijk et al., 2024).

On the other hand bacteria can exhibits form of self-defence, studies suggest that the mutation rate changes can occur in bacterial cells at higher temperatures. Based on an analysis of mutation accumulation experiments, and subsequent fitness evaluations of *E. coli* cells, it has been observed that, while the overall mutation rate remains similar across different temperatures, the proportion of beneficial mutations increases with rising temperatures (Chu et al., 2020). This suggests that feverish conditions might favor the rapid emergence of bacteria with better survivability abilities (MacFadden et al., 2018; Chu et al., 2020). As mutations are undirected the result of that study may suggest some way of bacterial form of protection from fever-like temperatures. Another important question concerns how gradual versus rapid temperature changes within the human fever range influence bacterial mutations, which allows them to survive.

### 9.3 Bacterial thermotolerance related to quorum sensing and biofilm formation

Elevated temperatures within the fever-like degree can significantly modulate the processes of biofilm formation and quorum sensing processes, which are crucial for bacterial survival and host colonization (Roncarati et al., 2025). During fever, biofilm plays a crucial role in bacterial survival, allowing them to remain hidden and protected from the host immune system. Studies suggest that due to their density and other properties, such as species-specific biofilm composition, some antibiotics may have reduced affinity for certain bacteria (Donlan and Costerton, 2002; Moser et al., 2017; Vestby et al., 2020).

Besides the protective role of bacterial biofilms, they can also cause damage to nearby host cells and tissues. Biofilms can often induce a local inflammatory response, which may contribute to febrile symptoms or tissue damage and may even be linked to the initiation or progression of certain cancers, such as colorectal cancer (Moser et al., 2017; Vestby et al., 2020). Furthermore, the physical presence of biofilm can work as a host place for other bacteria species, accumulating diverse bacterial populations and potentially enhancing their collective virulence through synergistic interactions (Vestby et al., 2020).

The relationship between these factors has been extensively investigated particularly in the context of wound infections, which can be accompanied by elevated temperatures caused by an inflammatory response. Analysis of biofilm composition by PCR shows that it contains a wide variety of bacterial species, differing from each other inside and outside the wound. The outer layers contain species such as *Staphylococcus aureus* and *Pseudomonas aeruginosa*, while the inner layers contain more anaerobic species, more resistant to oxygen deprivation (James et al., 2008; Vestby et al., 2020). Analysis of cocultures with *Pseudomonas aeruginosa* and *Staphylococcus aureus* indicates that the bacteria in cocultures have reduced motility (as evidenced by plate motility assays) and biofilm formation capacity (as determined by RNA-seq), but this is slightly increased at febrile temperatures (Solar Venero et al., 2024).

Bacteria biofilm also behave differently when heated. Bacteria from the *Vibrio* family exhibit increased biofilm formation at optimal temperatures around 25 °C, followed by a decrease at lower and higher temperatures, from 15 °C to 37 °C (Song et al., 2017). On the other hand *S. aureus* demonstrate enhanced biofilm formation at temperature that ranges up till 39 °C followed by rapid decline (Solar Venero et al., 2024), also *Klebsiella pneumoniae* ST307 (**Table 2**) shows enhanced biofilm formation at the temperatures around 37 °C. Those observations lead to a conclusion, that biofilm reflects the bacteria form of defenses from the excessive heating lower fever temperature, but decreases as the temperature rises above it. It might suggest that decreasing the temperature helps bacteria thrive, while taking into consideration biofilm formation.

### 9.4 Thermotolerance mechanisms by managing metabolic flux

Bacteria have evolved distinct strategies to meet this energy demand, which differ depending on whether the event is acute heat shock or long-term adaptation. In acute heat shock, cells prioritize survival over proliferation, initiating a rapid reallocation of resources. There are many *in vitro* studies that allow us to measure the effect of heat stress on the regulation of bacterial metabolism in isolated conditions.

Managing this acute crisis involves two main strategies. First, some bacteria, such as *Listeria monocytogenes*, when subjected to a heat shock of 48 °C *in vitro* (3 to 40 minutes), accelerate energy production by massively activating genes responsible for carbohydrate transport and metabolism. This, in turn, supplies the cell with ATP to fuel repair processes, while genes responsible for translation are simultaneously silenced to conserve resources and inhibit the synthesis of non-essential proteins (van der Veen et al., 2007). Second, in an ATP redirection strategy, *Staphylococcus aureus* exposed for 10 minutes to the lethal temperature of 48 °C *in vitro* maintains a constant intracellular ATP level despite the enormous energy demands of the protein quality system. This is achieved by inhibiting other major ATP-consuming pathways. Fleury et al (Fleury et al., 2009). observed a significant reduction in the expression of genes responsible for purine and pyrimidine synthesis (inhibiting DNA/RNA synthesis) and most aminoacyl-tRNA synthetases (reducing protein synthesis), effectively redirecting the existing ATP budget to functions essential for survival. This acute reprogramming can create metabolic imbalances. *In vitro* studies shows, that in *Escherichia coli* at 42 °C, a drop in the adenylate energy charge accelerates glycolysis. However, with the tricarboxylic acid (TCA) cycle simultaneously repressed, this leads to a significant accumulation of pyruvate. This results in the excretion of metabolites like acetate, and forces the cell to activate compensatory pathways, such as the glyoxylate shunt, to maintain essential anaplerosis (Wittmann et al., 2007). In contrast to acute shock, bacteria that undergo adaptive laboratory evolution experiment for thermotolerance exhibit a permanent, balanced reprogramming of their metabolism. A heat-adapted *Enterococcus faecium* strain, evolved for high-temperature survival, displays a slower growth phenotype (Wang et al., 2023a). This leads to the establishment of a new homeostatic state to face the harsh crisis conditions. This strain exhibits significantly decreased activity in its primary fermentative pathway by lactic dehydrogenase, and a marked downregulation of the TCA cycle and purine metabolism (Wang et al., 2023b). This evolved strategy permanently reallocates resources away from rapid growth and high-energy throughput, redirecting them toward constitutive reinforcement of cellular structures, such as the cell envelope.

Beyond broad energy management, thermotolerance involves specific adjustments to key metabolic networks and the accumulation of protective small molecules. The response of the TCA cycle is highly species-related. An *in vitro* study on *E. coli* shifting from 30 °C to 45 °C (from 10 min up to 235 min) revealed an initial accumulation of TCA intermediates, such as fumarate and malate, suggesting heightened flux to maximize ATP generation (Jozefczuk et al., 2010). Similarly, *S. aureus* at a fever-like temperature of 39 °C for 2 h upregulates TCA cycle genes (Solar Venero et al., 2024). Conversely, under different stress conditions or in adapted strains, the TCA cycle is repressed to conserve resources (Wittmann et al., 2007; Wang et al., 2023b). Amino acid pools are fundamentally altered. *E. coli* demonstrates a significant increase in free amino acids (e.g., alanine, glutamate, isoleucine, threonine, lysine, asparagine, phenylalanine), which serve as a ready supply of precursors for the rapid synthesis of heat shock proteins (Jozefczuk et al., 2010). In other contexts, amino acids are repurposed for energy; *S. aureus* funnels serine, glycine, and threonine into central metabolism to produce pyruvate, redirecting it from biosynthesis to energy generation (Fleury et al., 2009). A hallmark of thermotolerance is the accumulation of compatible solutes (osmoprotectants), which stabilize proteins and membranes against thermal denaturation (Welsh, 2000; Hasanuzzaman et al., 2013; Hussain Wani et al., 2013). For example, trehalose and proline were the most prominently accumulated metabolites in heat-stressed *E. coli*, serving as thermoprotectants (Jozefczuk et al., 2010). Heat-adapted *E. faecium* also accumulates trehalose-6-phosphate, a precursor of trehalose (Wang et al., 2023b). Similarly, glycine betaine (GB), another potent osmoprotectant, is accumulated via different mechanisms. *L. monocytogenes* acutely imports GB and L-carnitine via the SigB-dependent *opuC* ABC transporter (van der Veen et al., 2007). In contrast, the adapted *E. faecium* mutant downregulates the *OpuBD* export gene, effectively trapping and concentrating its internal GB supply (Wang et al., 2023b). Finally, polyamines represent another critical class of protectants. The heat-resistant *E. faecium* strain also showed increased levels of the polyamines putrescine and spermidine. As polycations, they stabilize negatively charged molecules like DNA and preserve membrane integrity (Wang et al., 2023b). In later-stage adaptation, *E. coli* also accumulates protective metabolites like cadaverine and enterobactin (Kim et al., 2020). Temperature shifts also function as environmental cues that modulate complex inter-species interactions. Solar Venero et al. investigated the response of *S. aureus* and *Pseudomonas aeruginosa* to a fever-like 39 °C for 2h in monoculture and 24h in co-culture. In monoculture, *S. aureus* exhibited a robust metabolic shift, while *P. aeruginosa* remained largely stable. However, in co-culture at 39 °C, the interaction intensified: *S. aureus* shifted strongly toward fermentation (producing lactate), and *P. aeruginosa* responded by upregulating genes for lactate oxidation, effectively cross-feeding on the *S. aureus* byproducts (Solar Venero et al., 2024). This demonstrates that metabolic adaptation to heat is not only species-specific but is critically influenced by the surrounding microbial community.

## 10 Discussion and outlook

**Figure 4:**
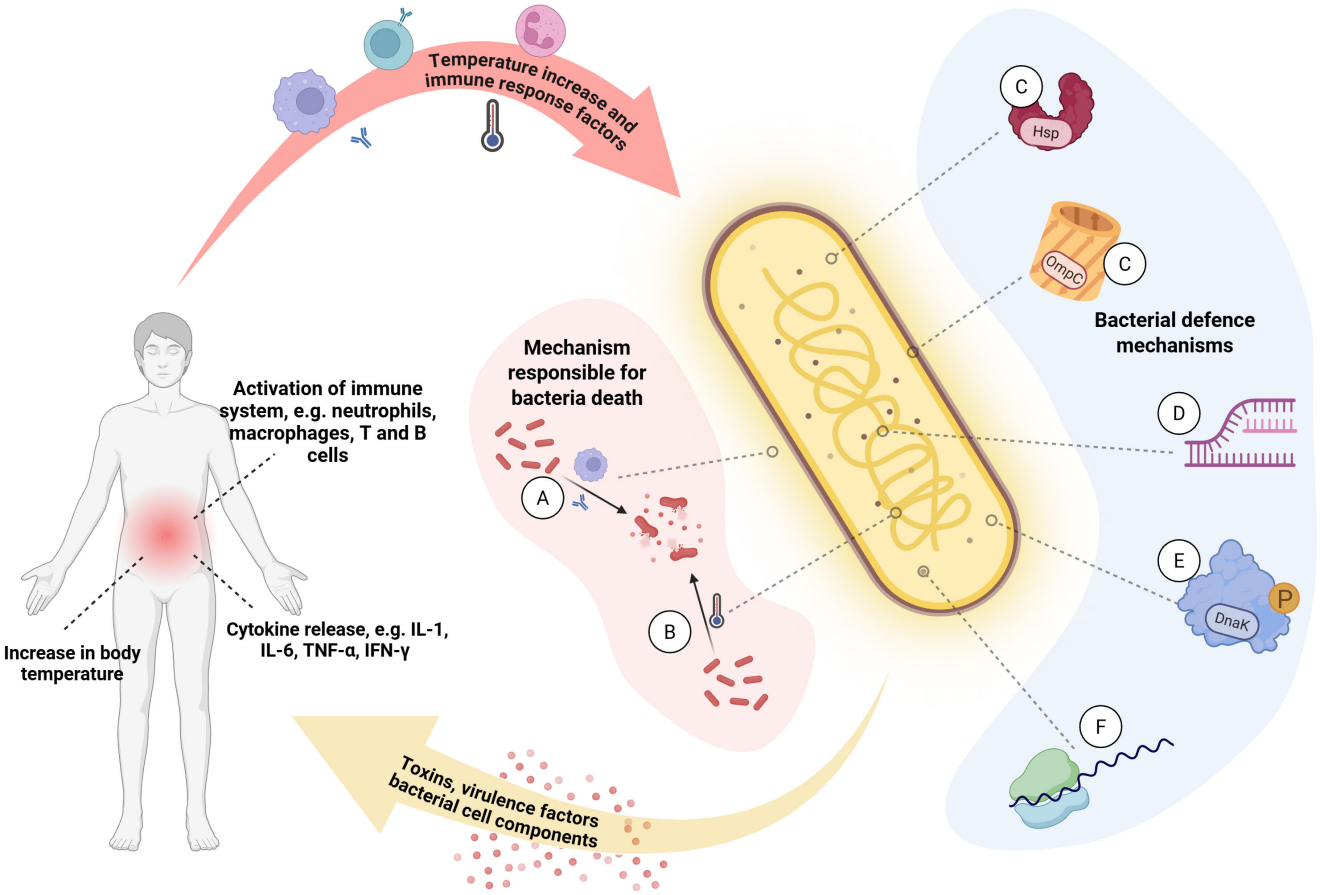
Host-pathogen interaction under fever development. Bacterial infection starts with the transfer of bacterial cells into the host organism, which triggers the immune response by activating immune cells, including macrophages and neutrophils and leads to the release of immune mediators, such as antibodies or cytokines **(A)**. Subsequently this process is accompanied by the release of bacterial antigens **(B)**, which prolongs the immune response and increases the temperature of the human body. These processes affect the bacterial cells and lead to pathogen death and the release of bacterial virulence factors, and cell components that may further induce the inflammation. Changing conditions related to temperature increase and immune system activation trigger bacterial mechanisms of adaptation, which allow them to survive in the host: **(C)** activation of heat response proteins HSP, which start the heat response cascade in bacterial cells; **(D)** regulation of membrane proteins expression e.g. OmpC, that hardens the cell membrane and makes it less permeable; **(E)** increased translation rates of various proteins e.g. virulence factors or inflammatory particles; **(F)** activation of multiple proteins e.g. chaperons that secure the bacterial genome from being damaged; **(G)** stabilized ribosomal proteins levels and their functions. Created in BioRender. Kramarczyk, K. (2026) https://BioRender.com/ap6ajuk.

Even though the current immunological, neurological, endocrine and metabolic pathways and the reasons involved in developing fever are known, there is still much information to be revealed about the mechanisms underlying the impact of fever on the process of microbial killing. Here, we have collected available information on the cycle of host-pathogen interactions, which occur under fever development. In our proposed cyclical model, we present the progression of infectious fever, which begins with the invasion of bacterial cells into the host. This is followed by the release and presentation of a series of external pyrogens, such as LPS. Extracellular pyrogens are responsible for the release of intracellular pyrogens in the host, such as cytokines, which subsequently trigger signaling pathways leading to both immune activation and increased body temperature. The increase in host body temperature leads to the initiation of defense mechanisms within the host and triggers similar mechanisms in bacterial cells exposed to heat stress (**Figure 4**).

The intrinsic effect of elevated body temperature on bacterial cells is poorly understood, although increasing the body temperature has been recognized as a therapeutic method for infectious diseases in thousands of cases. Literature shows that slightly elevated body temperature can damage and kill bacterial cells, but the mechanism underlying this process on the level of bacterial cells was never mechanistically described. The most intriguing concern about the efficacy of fever to harm pathogens is the febrile temperature, which rarely exceeds 40–41 °C in humans. In addition, several studies have shown that the temperature at different body compartments may vary significantly and can increase for 1-4 °C at the inflammation site (Maloney and Duffy, 2024), for instance, studies on inflamed atherosclerotic plaques have shown that their environments have temperatures up to 2 °C higher than the body core temperature (Casscells et al., 1996; Madjid et al., 2006). In addition, tissue-specific thermal gradients (Wang et al., 2025b) define how temperature fluctuates across various layers of biological material (e.g., skin, muscle, organs). They are heavily influenced by a tissue’s specific blood perfusion rate, metabolic activity, density, and thermal conductivity. This shows that the body temperature is highly variable and depends on the place, tissue or organ or the influence of external and internal factors. This raises the question, whether the heat of fever may harm or even kill the most severe human pathogens, which are residing in the inflammation site or if it might create them an easy target for the host immune system. An important observation from the Meltome Atlas studies shows that the proteins of some bacteria e.g. *E.coli* start to denature when the surrounding temperature exceed roughly 38-39 °C (Jarzab et al., 2020), which might point into the evolutionary features making the development of human thermoregulation designed to fight the infection altogether. This is also supported by the fact that pathogenic microorganisms find it easier to replicate and infect cells at temperatures below 37 °C (Geddes, 2020).

On the other side there are physiological processes happening in the host to fight the invading microorganisms under fever. A new study in mice has already shown that it helps immune cells more quickly reach and attack harmful germs. The authors showed that the heat shock proteins 90 (Hsp90)-induced α-integrin activation and signaling in T cells, facilitates their adhesion and transmigration (Lin et al., 2019). Also, the immune cells, like blood mononuclear cells activated by IL-2 or IFN-γ rapidly generate heat and may be suppressed by IL-10, which plays a role as a modulator of heat release under infection (Nogueira-MaChado et al., 1999). This happens probably due to immune cells, including neutrophils, macrophages and lymphocytes, which are recruited to the infection site and participate in heat generation. Therefore, it is not surprising that the optimal functional temperature of activated leukocytes is higher than normal core body temperature. Furthermore, this leads to the conclusion that raising slightly the temperature of a bacteria’s environment can lead to its death due to better recognition by a larger number of immune cells. Very likely fever provides a crucial temperature boost to locally warmed tissues at infected sites, elevating the temperature to the level that damages and kills pathogens (Evans et al., 2015). However, the exact temperature to which pathogens at the infection site are actually exposed is currently unknown and might be an interesting area for further exploration.

On one side microbes very rapidly respond and adapt to feverish conditions, carrying out adaptation processes that allow them to survive in host organisms. To some circumstances this might be also affected by long-term environmental warming, which significantly alters host-pathogen dynamics by pushing environmental pathogens closer to their thermal limits and shifts the baseline temperatures that trigger virulence regulation. These environmental shifts threaten to diminish the efficacy of host febrile responses and alter how pathogens adapt to thermal stress and alters the epidemiology of infectious diseases (Roncarati et al., 2025). On the other hand fever is an evolutionary adaptation of the host organism to fight the infection and it has been reported to have a cytotoxic effect on pathogens, inducing cellular stress and facilitating their damage. Synergistic action of stress factors such as higher temperature, combined with the antibiotic treatment, enhances immune response to a pathogen attack (Wrotek et al., 2021). This always leaves us with the questions, whether we should treat fever at the beginning of the infection or we should let it run under some circumstances. Most fever reducing drugs are given to patients with mild infections to improve their comfort but also due to general stress when the fever occurs. If fever reaches too high temperatures it may denature the body’s own proteins and alter normal cell metabolism, leading to cell injury and death. The same is associated with persistent high body temperature, which can trigger apoptosis. Treatments for severe fevers include anti-pyrogenic agents (egg. acetaminophen, ibuprofen) or aspirin, which also helps to stop blood clots that may coincide with severe fever. Although fever is not usually the direct cause of death in most cases, our new understanding of what happens at the level of bacterial cells may help us develop better methods of fighting infection entirely. However, beyond the unquestionable power of fever, many fundamental questions remain unresolved. Better understanding of the antibacterial effect by pointing to specific mechanisms of action elucidated under fever development might revolutionize standard thinking and demonstrate that lowering body temperature in mild infections may not be fully beneficial in the long lasting perspective and should always be balanced with high consideration.
